# Multimedia Exploration of a Complication Caused by a “Rogue” Barbed Suture

**DOI:** 10.1007/s11695-023-06735-8

**Published:** 2023-07-27

**Authors:** Mohamed Hany, Ahmed Zidan, Anwar Ashraf Abouelnasr, Mohamed Ibrahim, Bart Torensma

**Affiliations:** 1 Department of Surgery, Medical Research Institute, Alexandria University, 165 Horreya Avenue, Hadara, Alexandria, 21561 Egypt; 2 Madina Women’s Hospital, Alexandria University, Alexandria, Egypt; 3 Leiden University Medical Center (LUMC), Leiden, The Netherlands

**Keywords:** Revision Surgery, One Anastomosis Gastric Bypass, Barbed Sutures, Kinking

## Introduction

Barbed sutures have become an integral component in bariatric metabolic surgery (BMS) practices. This report details a case involving reversal of a one anastomosis gastric bypass after severe bowel dysfunction symptoms and significant weight reduction. A partial obstruction and kinking of the small bowel were found postoperatively to be consequent to the adherence of the barbed suture to the bowel mesentery. We highlight the approach and actions undertaken during the surgical intervention.

## Presentation and Pre-Workup

In 2023, a 42-year-old female patient presented to Madina Women’s Hospital, Alexandria University, Egypt, with severe bowel dysfunction symptoms, including diarrhea, steatorrhea, and notable muscle mass depletion. Her medical history revealed that the patient underwent a laparoscopic one anastomosis gastric bypass (OAGB) procedure in June 2021 at our center, at which point her body mass index (BMI) was recorded as 41.4 kg/m^2^, corresponding to a weight of 106 kg. Over the ensuing year, the patient experienced significant weight reduction, amounting to 58 kg, which resulted in a BMI of 18.75 kg/m^2^. Diabetes mellitus (type 2) was resolved by surgery.

Following a comprehensive evaluation by a multidisciplinary team (MDT) composed of a surgeon, nutritionist, and psychiatrist, the patient underwent a thorough preoperative workup, including imaging and endoscopic studies. Upon imaging and endoscopic results assessment, no abnormal findings were observed.

In addressing the patient’s symptoms, a treatment protocol was instituted, consisting of rifaximin to address small intestinal bacterial overgrowth, imodium to modulate bowel movement frequency and mitigate diarrhea, and creon to augment food digestion. Nevertheless, the effectiveness of this strategy was undermined by the patient’s failure to adhere to the prescribed medication regimen. In light of the persistent symptoms and after numerous consultations with the MDT, the patient requested to proceed with a procedure to restore her normal anatomical gastrointestinal pathway, thereby reversing the OAGB. A comprehensive discussion was undertaken regarding the potential ramifications of restorative BMS, and the possibility of weight regain and recurrence of medical associated problems, and the patient provided informed consent for the revision surgery. All preoperative laboratory investigations were within normal limits.

### Revision Surgery

During the revision surgery, we successfully restored the gastric pouch and normal anatomical pathway of the small bowel. The bowel was subsequently oversewn with barbed sutures (absorbable PDS V lock 3.0, Covidien, Mansfield, MA, USA), and the postoperative course was uneventful. No drain was inserted.

### Postoperative

On the fifth postoperative day, the patient reported abdominal discomfort in distension and nausea, culminating in vomiting. Abdominal examination showed mild tenderness with exaggerated bowel sounds. Despite normal laboratory findings and vital signs, the computed tomography (CT) scan with oral contrast revealed dilated small bowel proximal to the site of the previously anastomosed intestine with the passage of the dye distal to this site. Thereby, conservative therapeutic measures were taken. These measures, including cessation of oral intake, initiation of intravenous nutrition, and administration of gastrografin, however, did not yield the desired improvement in the patient’s condition. As vomiting and colics became frequent with no passage of flatus or stools, laparoscopic exploration was decided 2 days after admission.

Subsequently, a re-laparoscopic exploration was executed, revealing a dilatation of the small bowel proximal to the site of previous anastomosis, and kinking was found due to the adherence of the barbed suture to the mesentery. After untwisting the kink and trimming the large, barbed suture from the mesentery, the bowel was carefully examined, revealing an unaltered anatomical structure. The patient’s postoperative course proceeded without further complications. On day 1, after re-exploration, the bowel discomfort disappeared, nausea and vomiting were absent, and she had diarrhea several times, tolerating oral intake, and was ultimately discharged without residual symptoms on the third postoperative day. CT was done before discharge, and small bowel dilatation improved with the normal dye flow.

## Discussion

The OAGB is the third most common BMS procedure globally and is widely acknowledged for its effectiveness and reliability [1]. A systematic review by Khrucharoen et al. on revisional surgery after OAGB revealed that the transition to Roux-en-Y gastric bypass (RYGB) was the most frequently performed revisional operation. The second most common revisional approach following OAGB was a return to the original anatomical structure. Reasons for revision are bile reflux, malnutrition, bowel symptoms, or significant weight loss [2].

### Rogue Barbed Suture

During the use of barbed suture, when the cut end is left long, the barbed suture can become rogue, whereby it could attach to the underlying small bowel or mesentery. This attachment could instigate kinking and create a point of obstruction. Furthermore, the small bowel could twist around this fixation point, leading to volvulus with decreased mesenteric blood flow and ischemia as a consequence. In the literature, there are reports of obstruction following the utilization of barbed sutures in several disciplines but not described in BMS. Despite its apparent rarity with barbed sutures, a similar phenomenon has been reported infrequently in a case study. A “rogue staple” was present. An adjacent structure led to small bowel obstruction within the early postoperative period [3].

While such complications are rare, they are highly consequential when they do occur. Since the introduction of barbed sutures, and small randomized control trials with limited statistical power showed positive results for the barbed suture [4], the probability of encountering a complication is small, and the implications can be significant, as described in this multimedia article.

These findings underscore the importance of careful handling and judicious application of barbed sutures and the potential risk in BMS procedures.

Furthermore, this is the first visual documentation providing a practical context to the textual literature, offering an additional perspective on the potential complications of barbed sutures in real-world surgical settings.

## Conclusion

The barbed suture could contribute to or aggravate an existing problem and, therefore, should be trimmed short or buried using absorbable sutures to prevent long-thread suture complications.

## Supplementary information

Below is the link to the electronic supplementary material.

Online Resource 1 (MP4 227 MB)

## Data Availability

All data is available at the corresponding author.
